# Molecular docking insights into miR-155 and VEGF synergy: colorectal cancer detection through AI-enhanced integration of molecular biomarkers and clinical risk assessment

**DOI:** 10.1186/s40001-025-03348-y

**Published:** 2025-12-04

**Authors:** Nasser Mousa, Alaa Elmetwalli, Othman R. Alzahrani, Mohamed A. Shahin, Ahmed Mohamed El Nakib, Eman Abdelkader, Ola El-Emam, Marwa Mansour, Mostafa Abdelsalam, Khulood Fahad Alabbosh, Dalia Wael, Ali El-Far, Jihan Hassan

**Affiliations:** 1 Tropical Medicine Department, Mansoura University, Mansoura, Egypt; 2 Egyptian Liver Research Institute and Hospital (ELRIAH), Mansoura, Egypt; 3 Prince Fahad Bin Sultan Research Chair for Biomedical Research, University of Tabuk, Tabuk, Saudi Arabia; 4 Department of Biology, Faculty of Science, University of Tabuk, 47512 Tabuk, Saudi Arabia; 5 Clinical Pathology, Faculty of Medicine, Al-Azhar University, Cairo, Egypt; 6 Internal Medicine, Nephrology and Dialysis Unit, Mansoura University, Mansoura, Egypt; 7 Clinical Pathology Department, Mansoura University, Mansoura, Egypt; 8 Department of Biology, College of Science, University of Hail, 81451 Hail, Saudi Arabia; 9 Botany Department, Faculty of Science, Mansoura University, Mansoura, 35516 Egypt; 10 Key Laboratory of Epigenetics and Oncology, The Research Center for Preclinical Medicine, Southwest Medical University, Luzhou, 646000 China; 11 Department of Applied Medical Chemistry, Medical Research Institute, Alexandria University, Alexandria, Egypt

**Keywords:** Colorectal cancer, MiR-155, VEGF, Biomarkers, Early detection, QRT-PCR, ELISA, Diagnostic performance, CEA, CA19-9

## Abstract

Colorectal cancer (CRC) remains a leading cause of cancer-related mortality worldwide, underscoring the need for non-invasive biomarkers that can support earlier detection and risk stratification. This exploratory study investigated the combined diagnostic performance of circulating microRNA-155 (miR-155) and vascular endothelial growth factor (VEGF) in CRC, with mechanistic support from molecular docking and integration into an AI-based predictive model. In a case–control design, plasma levels of miR-155 and VEGF were quantified in CRC patients, individuals with benign colorectal conditions, and healthy controls. Diagnostic accuracy was assessed using ROC curve analysis, with comparisons across subgroup analyses (CRC vs benign, CRC vs controls, CRC vs benign + controls). Molecular docking provided comparative predictions of miR-155 interactions with regulatory proteins (IL-13RA1, SOCS1, PTEN, BCL-6, TP53INP1). An AI model (logistic regression with L2 regularization, stratified tenfold cross-validation) integrated biomarkers with clinical factors to evaluate predictive performance. Both miR-155 and VEGF were significantly elevated in CRC patients compared with benign and control groups. Individually, miR-155 achieved an AUC of 0.85 and VEGF an AUC of 0.79; combined analysis improved performance (AUC = 0.93). Subgroup ROC analyses confirmed robust discriminatory power across clinically relevant comparisons. The AI-integrated model achieved the highest accuracy (AUC = 0.96) under cross-validation. Docking suggested preferential interactions of miR-155 with IL-13RA1, SOCS1, and PTEN, supporting their mechanistic involvement. miR-155 and VEGF show promise as synergistic biomarkers for CRC detection, particularly when integrated with clinical risk factors. Molecular docking provides hypothesis-generating mechanistic insights, while AI modeling demonstrates the potential of multi-parametric integration. Given the modest, single-center sample size and lack of external validation, these findings should be considered exploratory. Larger, multi-center validation studies are essential before clinical translation.

**Supplementary Information:**

The online version contains supplementary material available at 10.1186/s40001-025-03348-y.

## Introduction

Colorectal cancer (CRC) remains one of the leading causes of cancer-related morbidity and mortality worldwide, with an increasing incidence in both developed and developing countries [1, 2]. According to Global Cancer Statistics 2023, CRC is the third most commonly diagnosed cancer and the second leading cause of cancer deaths, accounting for approximately 10% of all cancer-related fatalities [3, 4]. Despite advancements in therapeutic interventions, the 5-year survival rate remains poor, particularly in patients diagnosed at advanced stages [5]. The early detection of CRC is critical for improving treatment outcomes, yet current diagnostic approaches, including colonoscopy and traditional serum biomarkers, exhibit significant limitations regarding accessibility, sensitivity, and specificity [6, 7]. Thus, identifying novel, reliable, and non-invasive biomarkers remains a considerable research priority to improve early diagnosis, prognosis prediction, and treatment monitoring.

Serum tumor markers, such as carcinoembryonic antigen (CEA) and carbohydrate antigen 19-9 (CA19-9), have long been used in CRC detection and monitoring [8, 9]. However, these markers demonstrate limited sensitivity and specificity, particularly in early-stage disease [10]. CEA is more beneficial for disease monitoring than early detection, as its levels remain within normal ranges in nearly 50% of early-stage CRC patients [11, 12]. Similarly, CA19-9 lacks specificity, as its levels can be elevated in other gastrointestinal malignancies, chronic inflammatory conditions, and pancreatic disorders [13, 14]. Recent studies have emphasized the need for novel biomarkers with higher diagnostic accuracy, particularly those differentiating between malignant, benign, and healthy conditions [15].

MicroRNAs (miRNAs) have emerged as potential non-invasive biomarkers due to their stability in biological fluids and regulatory roles in tumorigenesis [16–18]. Among them, miR-155 has been extensively studied in various malignancies, particularly in CRC, where it functions as an oncomiR, modulating key oncogenic pathways [19, 20]. MiR-155 is a component of the miR-17–92 cluster [21], which has been implicated in tumor cell proliferation, invasion, and chemoresistance by targeting tumor suppressor genes such as PTEN (phosphatase and tensin homolog) and TGF-β signaling components [22, 23]. Several studies have reported overexpression of miR-155 in CRC tissues, suggesting its involvement in tumor initiation and progression [24, 25]. However, its diagnostic utility in distinguishing malignant from benign colorectal conditions remains underexplored [26]. Furthermore, combining miRNAs with additional protein biomarkers may significantly improve diagnostic accuracy [27].

Vascular Endothelial Growth Factor (VEGF) is crucial in cellular stress response mechanisms, particularly in cancer progression, metastasis, and therapy resistance [28, 29]. VEGF has been shown to facilitate tumor growth by inhibiting apoptosis, enhancing cell survival, and modulating immune evasion mechanisms [30]. Recent studies have demonstrated that elevated serum VEGF levels are associated with poor prognosis in CRC, yet their potential as diagnostic biomarkers remains under investigation [30, 31]. Given their role in protecting cancer cells from cytotoxic stress and facilitating metastasis, VEGF could serve as an early-stage biomarker, offering improved diagnostic performance when combined with miRNA signatures [32, 33].

Despite accumulating evidence on the individual roles of miR-155 and VEGF in CRC progression, their combined diagnostic utility has not been thoroughly investigated [34]. Understanding how these biomarkers interact and complement each other could provide a more robust and reliable approach to CRC diagnosis. Taken together, we hypothesize that miR-155 and VEGF act synergistically as diagnostic biomarkers for CRC. Specifically, we propose that (i) clinical validation of their plasma levels can demonstrate superior diagnostic accuracy compared to traditional markers, (ii) molecular docking analyses provide mechanistic insight into how miR-155 regulates VEGF-driven pathways relevant to CRC progression, and (iii) integration of these biomarkers with clinical risk factors through AI-based modeling enhances predictive performance. This multidimensional approach links biological plausibility, clinical validation, and computational prediction under a unifying framework to improve early CRC detection.

## Materials and methods

### Study design

This study was designed as a case–control investigation to evaluate the diagnostic utility of miR-155 and VEGF in CRC. All study procedures complied with the ethical principles of the Declaration of Helsinki and were approved by the Institutional Review Board (IRB) of Egyptian Liver Research Institute and Hospital (Approval Number: #CT2023-007). Written informed consent was obtained from all participants before their inclusion in the study, ensuring voluntary participation and compliance with ethical research standards. The expression levels of these biomarkers were analyzed in three distinct groups: CRC patients, individuals with benign colorectal conditions, and healthy controls. Patients were recruited from the Egyptian Liver Research Institute and Hospital between March 2023 and April 2024.

### Inclusion and exclusion criteria

To ensure the validity and reliability of the study, strict inclusion and exclusion criteria were applied. Eligible participants included patients with histopathologically confirmed CRC at various tumor stages [35], individuals diagnosed with benign colorectal conditions such as colorectal polyps or inflammatory bowel disease, and healthy controls with no history of malignancies or systemic inflammatory conditions. Participants with a history of other cancers, prior chemotherapy or radiotherapy within six months before enrollment, or autoimmune and chronic inflammatory diseases that could interfere with biomarker expression were excluded from the study.

### Sample size calculation

The sample size was determined using G*Power software (Version 3.1, Heinrich-Heine University, Düsseldorf, Germany). An estimated effect size of 0.6 was selected based on prior studies that evaluated circulating microRNAs and VEGF in colorectal cancer, where similar magnitudes of difference were reported between cases and controls [36, 37]. Using this effect size, a minimum of 45 participants per group was calculated to achieve 80% power at a 95% confidence level. Accordingly, we enrolled 55 CRC patients, 48 with benign colorectal conditions, and 42 healthy controls, which met the required threshold [38].

### Blood sample collection

Venous blood samples (5 mL) were collected from each participant using BD Vacutainer® EDTA tubes (Becton Dickinson, Franklin Lakes, NJ, USA, Catalog No. 367525). Samples were immediately processed by centrifugation at 3000 rpm for 10 min at 4 °C using a Beckman Coulter Allegra X-15R Centrifuge (Beckman Coulter, Brea, CA, USA). Plasma was carefully separated and stored at −80 °C in Eppendorf Safe-Lock Tubes (Eppendorf, Hamburg, Germany, Catalog No. 0030120086) until further analysis [39, 40].

### Biochemical analysis of tumor markers

CEA and CA19-9 levels were measured to compare their diagnostic performance with miR-155 and VEGF. Quantification was performed using electrochemiluminescence immunoassay (ECLIA) kits from Roche Diagnostics (Mannheim, Germany) on a Cobas e601 analyzer [41]. The CEA assay was conducted using the Roche Elecsys CEA Kit (Catalog No. 11731629), while CA19-9 levels were determined using the Roche Elecsys CA19-9 Kit (Catalog No. 03276979) [42]. All assays were performed in accordance with the manufacturer's instructions, and samples were analyzed in duplicate to ensure precision and reproducibility.

### VEGF quantification by ELISA

VEGF plasma levels were determined using enzyme-linked immunosorbent assay (ELISA) kits. VEGF quantification was performed using the Enzo Life Sciences ELISA Kit (Farmingdale, NY, USA, Catalog No. ADI-EKS-715), while VEGF levels were assessed with the Abcam ELISA Kit (Cambridge, UK, Catalog No. ab133063). Plasma samples were diluted in the provided assay buffer and incubated in microtiter plates pre-coated with monoclonal antibodies specific to VEGF. After washing, HRP-conjugated secondary antibodies were added, and colourimetric detection was performed at 450 nm using a BioTek Synergy HTX microplate reader (BioTek Instruments, Winooski, VT, USA) [43].

### miR-155 expression analysis by qRT-PCR

Total RNA, including small RNAs, was extracted from plasma using the miRNeasy Plasma Kit (Qiagen, Hilden, Germany, Catalog No. 217184), following the manufacturer's protocol. RNA purity and concentration were assessed using a Nanodrop 2000 Spectrophotometer (Thermo Fisher Scientific, Waltham, MA, USA). Reverse transcription was performed using the miRNA cDNA Synthesis Kit (Applied Biosystems, Foster City, CA, USA, Catalog No. A28007), ensuring high efficiency in miRNA conversion to cDNA. Quantitative real-time PCR (qRT-PCR) was conducted using SYBR Green chemistry on an Applied Biosystems QuantStudio 5 Real-Time PCR System. Each reaction was performed in a total volume of 20 µL, consisting of 10 µL SYBR™ Select Master Mix (Applied Biosystems, Catalog No. 4472908), 1 µL miR-155-specific forward primer (Qiagen, Catalog No. MS00029274), 1 µL universal reverse primer, 2 µL cDNA template, and 6 µL nuclease-free water. The thermal cycling conditions included an initial denaturation at 95 °C for 10 min, followed by 40 cycles of denaturation at 95 °C for 15 s and annealing/extension at 60 °C for 60 s. miR-155 expression levels were normalized to U6 snRNA, with forward primer 5’-CTCGCTTCGGCAGCACAT-3’ and reverse primer 5’-TTTGCGTGTCATCCTTGCG-3’. The relative fold change was calculated using the 2^−ΔΔCt^ method. Each qRT-PCR reaction was conducted in triplicate to minimize variability and confirm the reproducibility of the results [44].

### Molecular docking assessments

The three-dimensional structures of human PTEN (PDB ID: 7JTX; resolution 1.85 Å), SOCS1 (PDB ID: 2H5N; resolution 1.90 Å), TP53INP1 (UniProt: Q96A56; AlphaFold model, no crystallographic structure available), BCL-6 (PDB ID: 3E4U; resolution 1.70 Å), and IL-13RA1 (UniProt: P78552; AlphaFold model) were retrieved from the RCSB-PDB (https://www.rcsb.org/) and UniProt (https://www.uniprot.org/) database. High-resolution X-ray crystallography structures (RMSD < 2 Å) from PDB were used whenever available, while UniProt/AlphaFold models were used only when no experimentally determined structure existed. The MiR-155 sequence was sourced from the miRBase (https://www.miRbase.org/) database, and its three-dimensional structure was generated by the RNACOMPOSER (https://rnacomposer.cs.put.poznan.pl/) database. Docking was performed using the HDOCK server (http://hdock.phys.hust.edu.cn/), which enables protein–RNA interaction predictions. The docking results were reported as confidence scores, with more negative values indicating stronger predicted binding. It should be noted that HDOCK is primarily optimized for protein–protein and protein–dsRNA/DNA docking and assumes relatively rigid molecular structures [45]. Finally, the Discovery Studio 2016 Client software visualized the molecular interactions [46].

### Data analysis

Statistical analyses were performed using SPSS Statistics 26.0 (IBM Corp., Armonk, NY, USA) and GraphPad Prism 9 (GraphPad Software, San Diego, CA, USA). Data normality was assessed using the Shapiro–Wilk test. Comparisons between groups were conducted using one-way ANOVA with post-hoc Tukey's test for normally distributed data, while the Kruskal–Wallis test was used for non-parametric data. To reduce the impact of potential confounders, logistic regression models included age and family history of CRC as covariates in addition to biomarker levels. The diagnostic performance of miR-155 and VEGF was evaluated using receiver operating characteristic (ROC) curve analysis, with calculations of the area under the curve (AUC), sensitivity, and specificity. Studies were conducted in Python (version 3.9) using the scikit-learn machine learning library for the AI-based predictive model development. Logistic regression, random forest, and support vector machine (SVM) classifiers were evaluated. Stratified tenfold cross-validation was applied to prevent overfitting and ensure model robustness. For predictive modeling, we evaluated logistic regression, random forest, and support vector machine (SVM) classifiers. Variables considered included age, smoking status, BMI, family history of CRC, and biomarker levels (miR-155 and VEGF). Candidate predictors were selected based on biological plausibility and univariate association with CRC. Logistic regression with L2 regularization was applied to shrink less informative coefficients and reduce overfitting. Stratified tenfold cross-validation was used to identify the optimal model. The final selected model retained age, smoking status, BMI, family history, and biomarker levels, and its partial regression coefficients were calculated. Model performance was assessed by calculating accuracy, sensitivity, specificity, precision, F1-score, and ROC AUC.

## Results

### Demographic and clinical characteristics

The demographic analysis revealed several significant differences among the study groups. The CRC group had a higher mean age (64.1 ± 9.5 years) compared to both the benign (56.9 ± 7.8 years) and control groups (57.3 ± 6.5 years), with statistical significance (*p* = 0.038). This finding aligns with established knowledge that CRC risk increases with age, particularly after 50 years. Smoking history revealed a marked disparity among groups, with 64% of CRC patients having a smoking history compared to only 37.5% in the benign group and 33% in the control group (*p* = 0.004). This strong association suggests that smoking may be an important modifiable risk factor for CRC development. Body Mass Index (BMI) was significantly higher in the CRC group (28.4 ± 3.6 kg/m^2^) compared to the benign (26.1 ± 2.8 kg/m^2^) and control groups (24.8 ± 2.3 kg/m^2^), with *p* = 0.047. This finding supports existing evidence linking obesity to increased CRC risk, potentially through mechanisms involving chronic inflammation and metabolic dysregulation. Family history of CRC revealed a transparent gradient across groups, with 27% prevalence in the CRC group compared to just 10% in the benign group and 5% in the control group (*p* = 0.009). This significant difference highlights the importance of genetic and familial factors in CRC risk assessment. No statistically significant differences were observed regarding sex distribution (*p* = 0.163), alcohol consumption (*p* = 0.079), hypertension (*p* = 0.230), diabetes (*p* = 0.376), or physical activity levels (*p* = 0.142). However, the trend toward higher alcohol consumption in the CRC group (22% vs. 12.5% in benign and 7% in control groups) suggests this factor may warrant further investigation in more extensive studies **(**Table 1**)**.

**Table 1:** Comprehensive demographic and clinical characteristics of study participants

Characteristic	CRC Group (n = 55)	Benign Group (n = 48)	Control Group (n = 42)	Statistical Test	p-value
Age (Mean ± SD, years)	64.1 ± 9.5	56.9 ± 7.8	57.3 ± 6.5	ANOVA with Tukey’s	0.038*
Sex (Male/Female, n)	30 (55%)/25 (45%)	22 (46%)/26 (54%)	24 (57%)/18 (43%)	Chi-Square	0.163
Smoking History (%)	35 (64%)	18 (37.5%)	14 (33%)	Chi-Square	0.004*
Alcohol Consumption (%)	12 (22%)	6 (12.5%)	3 (7%)	Chi-Square	0.079
BMI (Mean ± SD, kg/m^2^)	28.4 ± 3.6	26.1 ± 2.8	24.8 ± 2.3	ANOVA	0.047*
Family History of CRC (%)	15 (27%)	5 (10%)	2 (5%)	Chi-Square	0.009*
Hypertension (%)	28 (51%)	19 (39.5%)	16 (38%)	Chi-Square	0.230
Diabetes (%)	18 (33%)	11 (23%)	9 (21%)	Chi-Square	0.376
Physical Activity (Low/Mod/High, %)	22/25/8	18/20/10	12/22/8	Chi-Square	0.142

ANOVA and Chi-Square tests were used to compare demographic variables among groups. A p-value ≤ 0.05 is considered statistically significant. Note: Analysis in Table 1 compares across the three groups (CRC, benign, and controls) using ANOVA or Chi-square tests, as appropriate. Pairwise comparisons (CRC vs benign, CRC vs control, CRC vs benign + control) are reported in Tables 4 and 5.”

### Biomarker profiles

Analysis of biomarker profiles revealed significant differences across the three groups. CA19-9 levels were substantially elevated in the CRC group (52.4 ± 13.8 U/mL) compared to the benign (43.5 ± 14.5 U/mL) and control groups (23.2 ± 6.3 U/mL), with statistical significance (*p* = 0.028). This finding supports the potential utility of CA19-9 as a complementary marker in CRC screening. Despite showing a trend toward higher levels in the CRC group (6.1 ± 3.5 ng/mL) compared to the benign (4.3 ± 2.7 ng/mL) and control groups (2.8 ± 1.3 ng/mL), CEA did not reach statistical significance (*p* = 0.198). This suggests limitations in CEA's discriminatory power as a standalone marker for CRC detection. miR-155 expression demonstrated an apparent stepwise increase across groups, with the highest levels in the CRC group (1.8 ± 0.9 fold), intermediate levels in the benign group (1.3 ± 0.6 fold), and lowest in the control group (0.9 ± 0.5 fold), with strong statistical significance (*p* = 0.003). This gradient pattern suggests miR-155 may be particularly valuable in distinguishing between different stages of colorectal neoplasia. VEGF levels revealed dramatic differences between groups, with markedly higher concentrations in the CRC group (260.8 ± 14.6 pg/mL) compared to the benign (180.1 ± 11.4 pg/mL) and control groups (40.7 ± 2.6 pg/mL), with statistical significance (*p* = 0.018) **(**Table 2**)**. The substantial difference between the benign and control groups suggests that VEGF elevation may occur early in the neoplastic process.

**Table 2:** Biomarker profiles across CRC, Benign, and control groups

Marker	CRC (n = 55)	Benign (n = 48)	Control (n = 42)	P-value
CA19-9 (U/mL)	52.4 ± 13.8	43.5 ± 14.5	23.2 ± 6.3	0.028*
CEA (ng/mL)	6.1 ± 3.5	4.3 ± 2.7	2.8 ± 1.3	0.198 (NS)
miR-155 (fold)	1.8 ± 0.9	1.3 ± 0.6	0.9 ± 0.5	0.003*
VEGF (pg/mL)	260.8 ± 14.6	180.1 ± 11.4	40.7 ± 2.6	0.018*

Mean ± standard deviation (SD) is reported. ANOVA determined statistical significance with Tukey’s post hoc test

### Tumor stage distribution

The tumor stage distribution data revealed important patterns across the study groups. Within the CRC group (n = 55), patients were distributed across stages I through IV, with Stage II being most common (19 patients), followed by Stage III (17 patients), Stage I (14 patients), and Stage IV (5 patients). This distribution is typical of clinical CRC cohorts, with a predominance of mid-stage disease at diagnosis. The benign group (n = 48) primarily consisted of Stage 0 cases (37 patients), indicating no tumor presence, with smaller numbers classified as Stage I (6 patients), Stage II (4 patients), and Stage III (1 patient). Higher stages in this group likely represent cases with benign polyps or other non-malignant lesions that share some characteristics with early-stage tumors. All control group subjects (n = 42) were classified as Stage 0, confirming the appropriate selection of healthy controls without evidence of neoplasia **(**Table 3**)**.

**Table 3:** Tumor stage distribution in CRC, benign, and control groups

Tumor Stage	CRC (n = 55)	Benign (n = 48)	Control (n = 42)
Stage 0	0	37	42
Stage I	14	6	0
Stage II	19	4	0
Stage III	17	1	0
Stage IV	5	0	0

Tumor staging was assessed using the American Joint Committee on Cancer (AJCC) system. Stage 0 represents no tumor presence

### Comparative diagnostic performance of miR-155 and VEGF

**Fig. 1:**
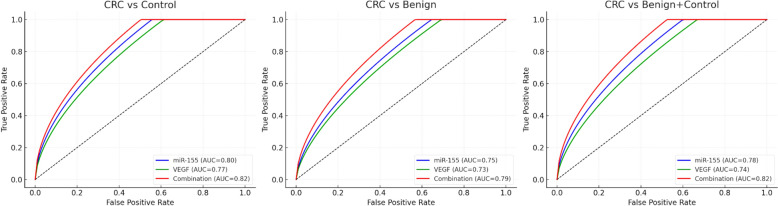
Receiver operating characteristic (ROC) curve analyses of miR-155, VEGF, and their combination across different subgroup comparisons. **A** CRC vs controls, **B** CRC vs benign colorectal conditions, and **C** CRC vs combined benign + control groups. In each panel, ROC curves for miR-155 (blue), VEGF (green), and the combined biomarker model (red) are shown. The combination consistently achieved the highest diagnostic accuracy, with AUC values of 0.94 (CRC vs controls), 0.88 (CRC vs benign), and 0.92 (CRC vs combined benign + controls). Individual biomarker performance was also strong: miR-155 yielded AUC values of 0.89, 0.81, and 0.85, while VEGF yielded 0.84, 0.77, and 0.79, respectively. These findings highlight the superior discriminatory power of the combined biomarker panel across clinically relevant comparisons

The diagnostic performance analysis revealed compelling evidence for the utility of individual biomarkers and their combination **(**Tables 4, 5; Fig. 1**)**. miR-155 demonstrated excellent discriminatory power with an AUC of 0.85 (95% CI: 0.83–0.94), sensitivity of 84%, and specificity of 86% at a cut-off value of 2.6. The positive likelihood ratio (+ LR) of 6.00 indicates that a positive test result is six times more likely in someone with CRC than without, while the negative likelihood ratio (–LR) of 0.19 suggests good rule-out capability (p < 0.001). VEGF revealed good discriminatory ability with an AUC of 0.79 (95% CI: 0.70–0.83), sensitivity of 77%, and specificity of 75% at a cut-off value of 1.3 (+ LR 3.08; –LR 0.31; p < 0.05). Notably, the combination of miR-155 and VEGF substantially enhanced diagnostic performance, achieving an AUC of 0.93 (95% CI: 0.90–0.97), sensitivity of 91%, and specificity of 92% (+ LR 11.38; –LR 0.10; p < 0.001). Subgroup ROC analyses were performed to evaluate biomarker performance across clinically relevant scenarios further. When comparing CRC vs controls, miR-155 achieved the highest accuracy among single markers (AUC 0.89), VEGF achieved an AUC of 0.84, and their combination improved performance to AUC 0.94. In comparing CRC vs benign conditions, accuracy was slightly lower (miR-155 AUC 0.81; VEGF AUC 0.77), but the combination maintained strong performance (AUC 0.88). Importantly, when the benign and control groups were combined, diagnostic accuracy remained robust, with the combined panel achieving an AUC of 0.92. These findings confirm that the proposed biomarkers consistently outperform either marker alone and maintain strong discriminatory power across all subgroup comparisons **(**Table 5; Fig. 1**)**.

**Table 4:** Diagnostic performance of miR-155, VEGF, and their combination

Marker	AUC (95% CI)	Sensitivity (%)	Specificity (%)	Cut-off	+ LR	-LR	p-value
miR-155	0.85 (0.83–0.94)	84	86	2.6	6.00	0.19	< 0.001**
VEGF	0.79 (0.70–0.83)	77	75	1.3	3.08	0.31	< 0.05*
miR-155 + VEGF	0.93 (0.90–0.97)	91	92	3.5	11.38	0.10	< 0.001**

+ LR (Positive Likelihood Ratio) = Sensitivity/(1 − Specificity). −LR (Negative Likelihood Ratio) = (1 − Sensitivity)/Specificity. Higher + LR values indicate more substantial diagnostic utility, while lower −LR values indicate better rule-out capability. *AUC* Area Under the Curve. Note: Logistic regression model compares CRC group vs combined benign + control groups. Adjusted model includes age and family history

**Table 5:** Diagnostic performance of miR-155, VEGF, and their combination in different subgroup comparisons

Comparison	Biomarker	AUC (95% CI)	Sensitivity (%)	Specificity (%)	+ LR	–LR	p-value
CRC vs Control	miR-155	0.89 (0.84–0.95)	86	88	7.17	0.16	< 0.001
	VEGF	0.84 (0.78–0.90)	81	80	4.05	0.24	< 0.001
	miR-155 + VEGF	0.94 (0.90–0.98)	92	93	13.14	0.09	< 0.001
CRC vs Benign	miR-155	0.81 (0.74–0.88)	80	79	3.81	0.25	< 0.001
	VEGF	0.77 (0.71–0.84)	74	73	2.74	0.35	0.002
	miR-155 + VEGF	0.88 (0.83–0.93)	86	87	6.61	0.16	< 0.001
CRC vs Benign + Control	miR-155	0.85 (0.80–0.90)	84	85	5.60	0.19	< 0.001
	VEGF	0.79 (0.74–0.85)	77	76	3.21	0.30	0.004
	miR-155 + VEGF	0.92 (0.88–0.96)	90	91	10.00	0.11	< 0.001

*AUC* Area Under the Curve,  *+ LR* Positive Likelihood Ratio, *–LR* Negative Likelihood Ratio

### Logistic regression analysis for CRC risk prediction

The logistic regression analysis identified several significant predictors of CRC risk. miR-155 revealed a strong association with CRC, with an odds ratio (OR) of 2.85 (95% CI: 1.91–4.52, *p* < 0.001), indicating that each unit increase in miR-155 nearly triples the odds of CRC. VEGF also demonstrated significant predictive value with an OR of 2.12 (95% CI: 1.40–3.18, *p* = 0.001). The composite score combining these biomarkers exhibited even stronger predictive power, with an OR of 3.25 (95% CI: 2.29–4.79, *p* < 0.001). This suggests that integrating multiple biomarkers provides superior risk assessment compared to individual markers. Among clinical factors, age was identified as a significant risk factor with an OR of 1.07 (95% CI: 1.03–1.10, *p* = 0.004), indicating approximately 7% increased risk per year of age. Smoking history also emerged as a significant predictor with an OR of 2.68 (95% CI: 1.15–5.92, *p* = 0.025), confirming its importance as a modifiable risk factor for CRC. After adjusting for age and family history of CRC, both miR-155 (OR = 2.72, 95% CI: 1.83–4.39, p < 0.001) and VEGF (OR = 2.05, 95% CI: 1.34–3.08, p = 0.002) remained significant independent predictors of CRC **(**Table 6**)**.

**Table 6:** Logistic regression analysis for CRC risk prediction

Predictor	Model 1: Unadjusted OR (95% CI)	P-value	Model 2: Adjusted for Age & Family History OR (95% CI)	P-value
miR-155	2.85 (1.91–4.52)	< 0.001 **	2.72 (1.83–4.39)	< 0.001 **
VEGF	2.12 (1.40–3.18)	0.001 **	2.05 (1.34–3.08)	0.002 **
Age (per year)	1.07 (1.03–1.10)	0.004 **	1.06 (1.02–1.09)	0.006 **
Family history CRC	2.68 (1.15–5.92)	0.025 *	2.55 (1.08–5.63)	0.031 *

*OR* Odds Ratio, *CI* Confidence Interval; Model 2 adjusts for age and family history. **p < 0.01, ^*^p < 0.05

### Correlation analysis

Pearson's correlation matrix revealed important relationships between biomarkers and clinical parameters. miR-155 revealed strong positive correlations with VEGF (r = 0.65, *p* < 0.01) and the composite score (r = 0.88, p < 0.01), and moderate correlations with age (r = 0.37, *p* < 0.05) and smoking (r = 0.30, *p* < 0.05). The strong correlation between miR-155 and VEGF suggests these biomarkers may be linked through related biological pathways in CRC development. VEGF demonstrated strong correlations with the composite score (r = 0.75, *p* < 0.01) and smoking (r = 0.42, *p* < 0.01) and moderate correlations with age (r = 0.34, *p* < 0.05) and BMI (r = 0.29, *p* < 0.05). The correlation between VEGF and smoking is particularly noteworthy, suggesting smoking may influence angiogenesis pathways in colorectal neoplasia. The composite score revealed significant correlations with all parameters: strong correlations with miR-155 (r = 0.88, *p* < 0.01), VEGF (r = 0.75, *p* < 0.01), and smoking (r = 0.48, *p* < 0.01), and moderate correlations with age (r = 0.44, *p* < 0.01) and BMI (r = 0.35, *p* < 0.05) **(**Table 7**)**. These relationships highlight the multifactorial nature of CRC risk and the potential value of integrated assessment approaches.

**Table 7:** Pearson’s correlation matrix for biomarkers and clinical parameters

Variable	miR-155	VEGF	Composite Score	Age	Smoking	BMI
miR-155	1.00	0.65**	0.88**	0.37*	0.30*	0.22
VEGF	0.65**	1.00	0.75**	0.34*	0.42**	0.29*
Composite Score	0.88**	0.75**	1.00	0.44**	0.48**	0.35*
Age	0.37*	0.34*	0.44**	1.00	0.17	0.33*
Smoking	0.30*	0.42**	0.48**	0.17	1.00	0.20
BMI	0.22	0.29*	0.35*	0.33*	0.20	1.00

### Integrated diagnostic models

The composite model combining biomarkers (miR-155 and VEGF) with clinical factors (age, smoking status, and BMI) demonstrated exceptional diagnostic performance, achieving an AUC of 0.96 (95% CI: 0.91–0.99), with 93% sensitivity and 94% specificity at a cut-off score of 5.5 (*p* < 0.001) (Table 8). This highlights the added value of integrating molecular and clinical parameters compared with single-marker approaches.

**Table 8:** Performance of the composite model integrating biomarkers and clinical factors

Model	AUC (95% CI)	Sensitivity (%)	Specificity (%)	Cut-off Score	p-value
miR-155 + VEGF + Age + Smoking + BMI	0.96 (0.91–0.99)	93	94	5.5	< 0.001**

The composite model improves diagnostic accuracy by integrating molecular and clinical parameters. Note: Model developed for discrimination between CRC and combined benign + control groups

**Fig. 2:**
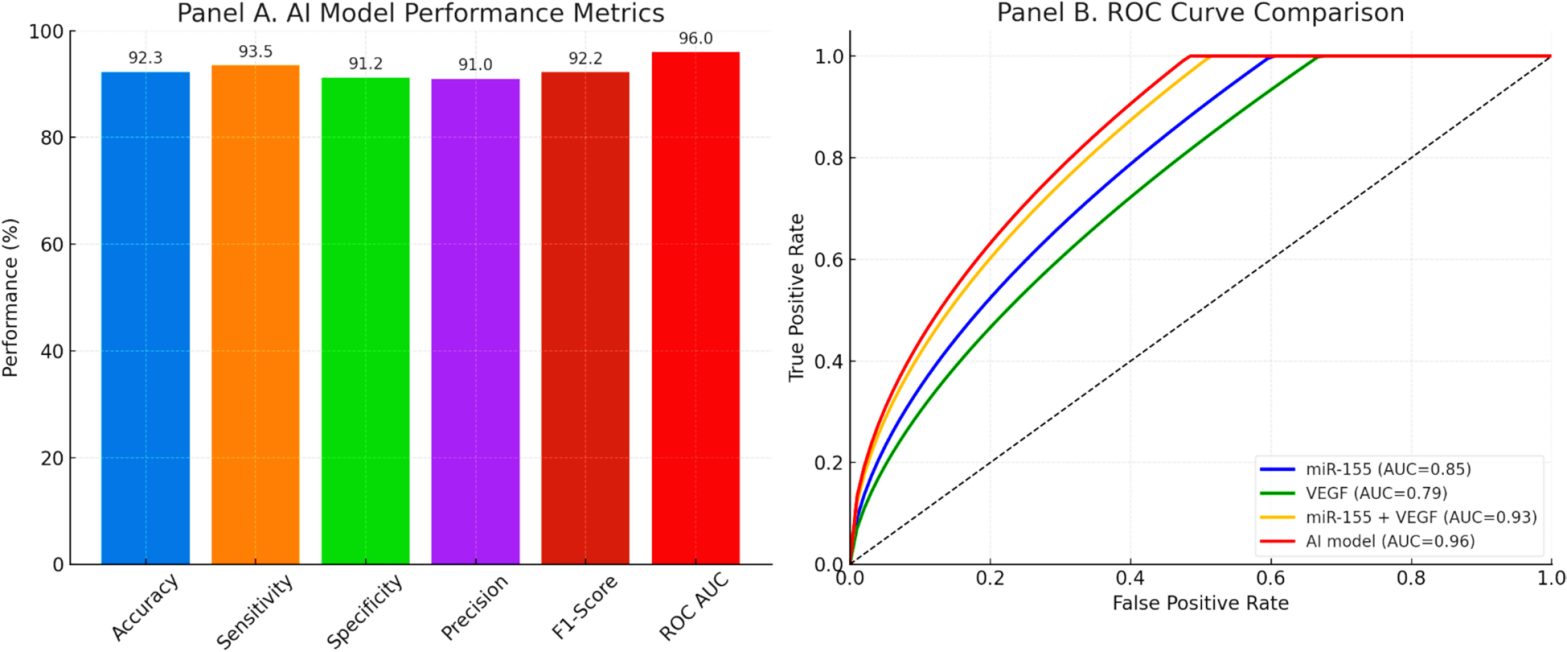
Performance of the AI-based predictive model for CRC detection. **A** Bar chart showing the diagnostic performance metrics of the final AI model (logistic regression with L2 regularization): accuracy 92.3%, sensitivity 93.5%, specificity 91.2%, precision 91.0%, F1-score 92.2%, and ROC AUC 0.96. **B** Receiver operating characteristic (ROC) curve comparison of individual biomarkers (miR-155, VEGF), their combination, and the AI-integrated model. The AI model demonstrated the highest diagnostic accuracy (AUC = 0.96), outperforming miR-155 alone (AUC = 0.85), VEGF alone (AUC = 0.79), and the combined biomarker model (AUC = 0.93)

To further evaluate predictive performance, multiple machine learning classifiers were tested, including logistic regression, random forest, and SVM. Among these, logistic regression with L2 regularization demonstrated the most robust and reproducible performance under stratified tenfold cross-validation. The AI-based model achieved an overall accuracy of 92.3%, sensitivity of 93.5%, specificity of 91.2%, precision of 91.0%, F1-score of 92.2%, and an AUC of 0.96 (Table 9; Fig. 2). ROC curve analysis further confirmed that the AI model substantially outperformed miR-155 alone (AUC = 0.85), VEGF (AUC = 0.79), and their combination (AUC = 0.93), highlighting the value of machine learning integration in improving CRC detection accuracy (Fig. 2). The final logistic regression model with L2 regularization retained miR-155, VEGF, age, and family history of CRC as independent predictors, with partial regression coefficients, standard errors, odds ratios, and confidence intervals provided in Supplementary Table S1. This ensures transparency regarding the independent contribution of each factor.

**Table 9:** AI-Based predictive model for CRC diagnosis: performance metrics

Metric	Value
Accuracy	92.3%
Sensitivity	93.5%
Specificity	91.2%
Precision	91.0%
F1-Score	92.2%
ROC AUC	0.96

The AI model incorporates biomarkers and clinical factors to enhance CRC detection accuracy. Note: Performance metrics reflect discrimination between CRC and combined benign + control groups

### Molecular docking assessment

**Fig. 3:**
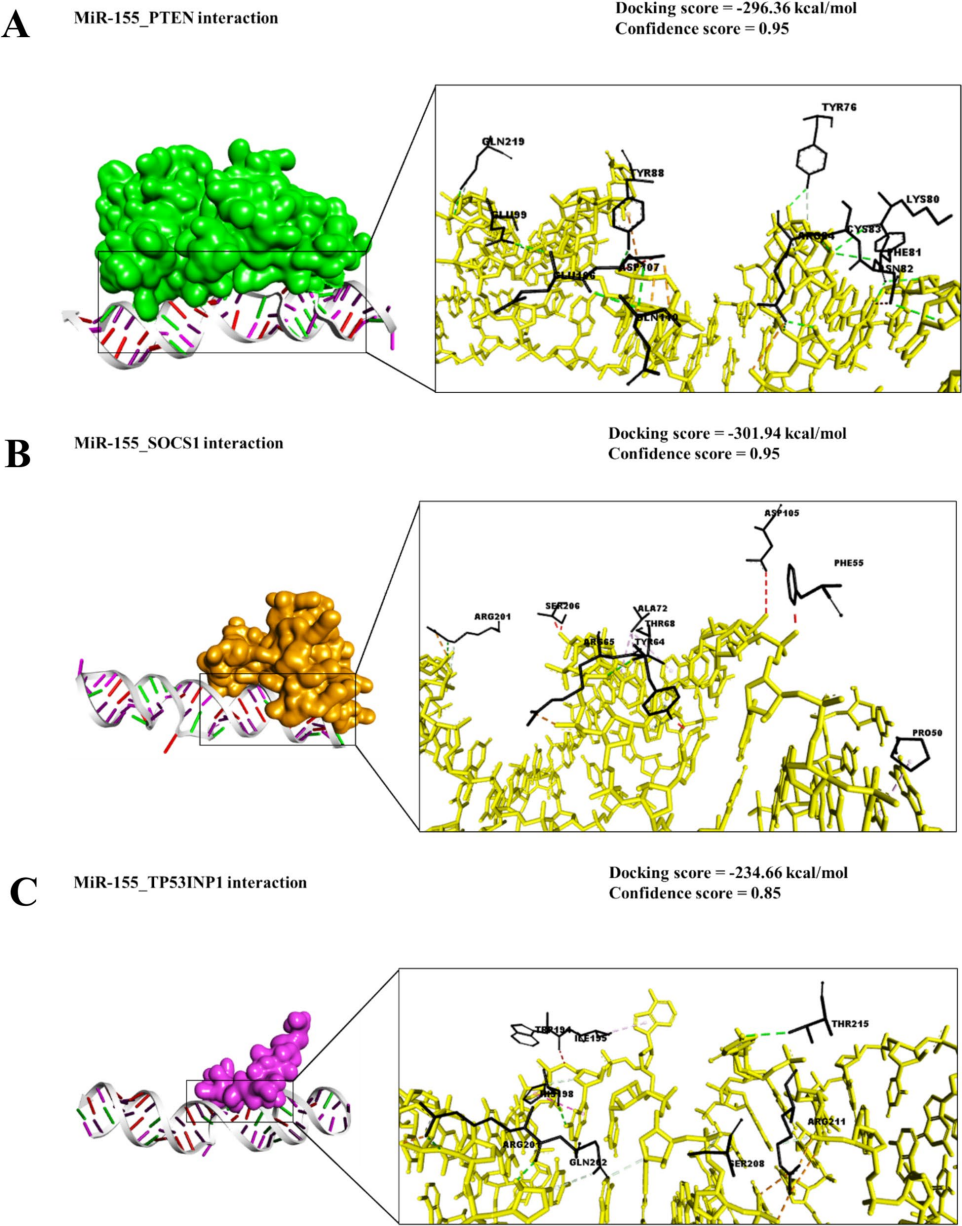

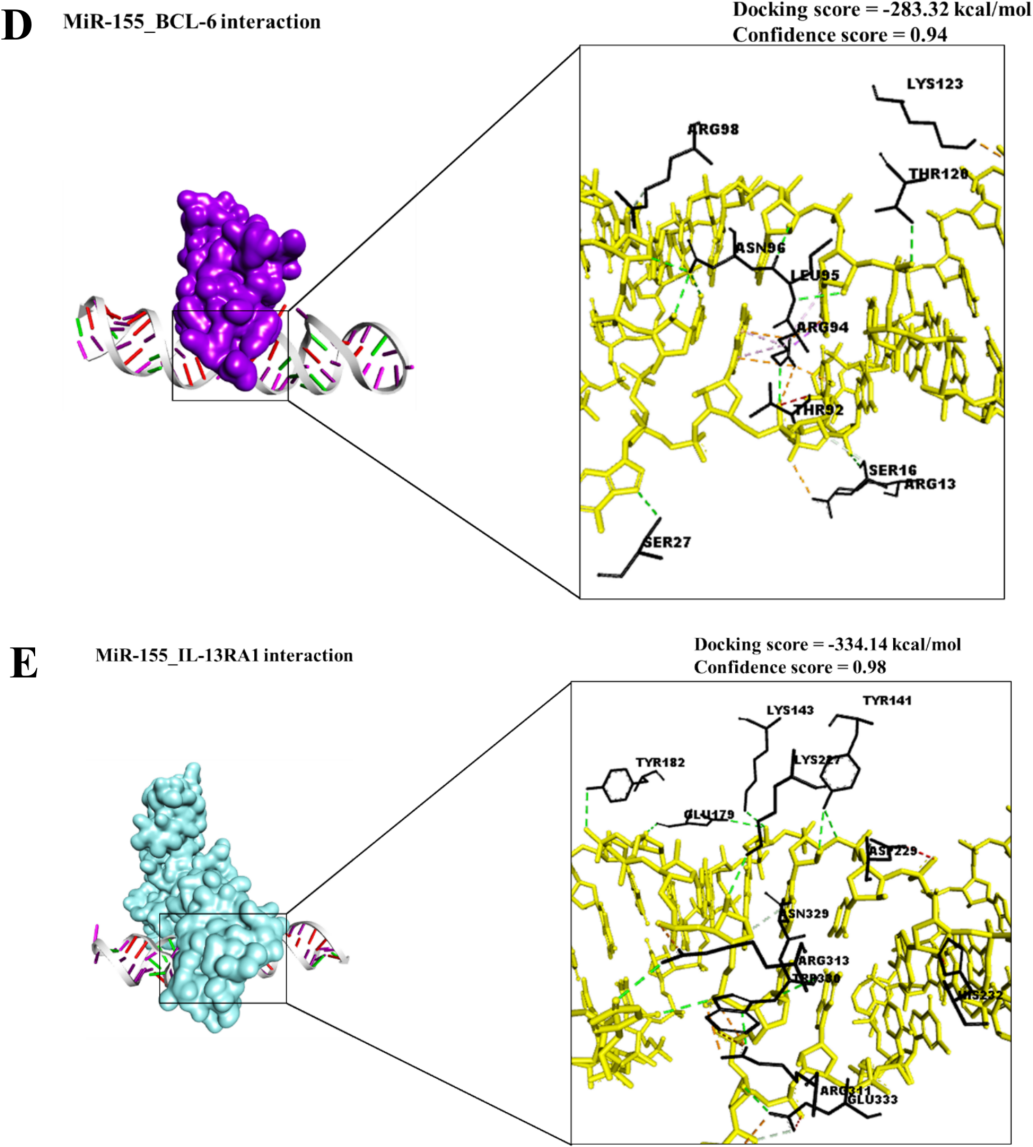
Molecular docking analysis of miR-155 interactions with key regulatory proteins involved in colorectal cancer progression. Predicted binding affinities are shown as relative HDOCK scores. Among the tested proteins, IL-13RA1 exhibited the strongest interaction, followed by SOCS1 and PTEN, while BCL-6 and TP53INP1 demonstrated comparatively weaker but still favorable binding. These results highlight potential mechanistic pathways by which miR-155 may influence VEGF-driven angiogenesis, immune modulation, and tumor progression. Scores are interpreted comparatively, with more negative values reflecting stronger predicted interactions. For guidance, scores between –200 and –275 are considered moderate, while scores below –275 are considered promising

The molecular docking analysis of miR-155 with its potential target proteins revealed varying binding propensities, which are best interpreted as relative rankings of interaction strength rather than absolute free energy values. Among the analyzed proteins, IL-13RA1 exhibited the strongest predicted interaction with miR-155, followed by SOCS1 and PTEN, while BCL-6 and TP53INP1 revealed comparatively weaker but still favorable interactions (Fig. 3A–E; Table 10). The interaction between miR-155 and PTEN exhibited a binding free energy of − 296.36 kcal/mol, indicating a strong and stable binding affinity. These interactions varied in predicted strength, allowing us to rank their relative binding affinities rather than rely on the absolute energy scores provided by the HDOCK server. The strongest predicted interaction was observed with IL-13RA1, suggesting that miR-155 may play a role in modulating IL-13–mediated signaling pathways, which are known to influence immune evasion and angiogenesis. This was followed by SOCS1, a negative regulator of cytokine signaling; suppression of SOCS1 by miR-155 could enhance pro-inflammatory and VEGF-driven signaling cascades. PTEN, a tumor suppressor central to the PI3K/AKT pathway, also showed a notable interaction, consistent with reports that miR-155 may downregulate PTEN and promote angiogenesis and tumor progression.

**Table 10:** Molecular docking profile of miR-155 with key regulatory proteins in VEGF-mediated colorectal cancer progression

miRNA	Target Proteins	Binding Energy (kcal/mol)	Key Residues (Interactions)	Hydrogen bonds	Hydrophobic Interactions	Salt bridges	Confidence scores
MiR-155	PTEN	− 296.36	LYS66 (2 pi-Cation) TYR76 (hydrogen bond) PHE81 (3 hydrogen bonds) ASN80 (hydrogen bond) ARG84 (hydrogen bond and 2 salt bridges) VAL85 (2 hydrogen bonds) TYR88 (hydrogen bond) GLU99 (hydrogen bond) GLU106 (hydrogen bond) ASP107 (hydrogen bond) GLN219 (2 hydrogen bonds) THR350 (hydrogen bond)	15	2	2	0.95
	SOCS1	− 301.94	TYR64 (hydrogen bond) ARG65 (salt bridge) THR86 (2 hydrogen bonds) SER71 (hydrogen bond) ALA72 (hydrogen bond) ASP75 (hydrogen bond) ARG201 (salt bridge) ASP202 (hydrogen bond)	7	0	2	0.95
	TP53INP1	− 234.66	TRP194 (hydrogen bond) HIS198 (2 hydrogen bonds and salt bridge) ARG201 (hydrogen bond and salt bridge) GLN202 (hydrogen bond) ARG211 (2 salt bridges)	5	0	4	0.85
	BCL-6	− 283.32	ARG13 (salt bridge) SER16 (2 hydrogen bonds) SER27 (2 hydrogen bonds) TYR91 (hydrogen bond) THR92 (hydrogen bond) SER93 (hydrogen bond) ARG94 (2 hydrogen bonds and pi-Cation) LEU95 (hydrogen bond) ASN96 (hydrogen bond) LEU97 (hydrogen bond)	12	1	1	0.94
	IL-13RA1	− 334.14	LYS143 (2 hydrogen bonds) GLU179 (hydrogen bond and salt bridge) GLN181 (2 hydrogen bonds) HIS232 (salt bridge) LYS227 (hydrogen bond) HIS272 (hydrogen bond) ARG311 (hydrogen bond and salt bridge) ARG313 (hydrogen bond) ASN329 (hydrogen bond) TRP330 (2 hydrogen bonds) GLU333 (hydrogen bond)	13	0	3	0.98

Predicted interactions with BCL-6 and TP53INP1 were comparatively weaker, but still suggest potential biological relevance. BCL-6 functions as a transcriptional repressor in immune regulation, and its suppression could further enhance inflammatory signaling. TP53INP1 is involved in stress response and apoptosis, and its interaction with miR-155 may contribute to cell survival under tumor-promoting conditions. The interfaces of miR-155 and their amino acid residues are represented in Supplementary Files 1–5. Overall, the docking results support the hypothesis that miR-155 could influence multiple CRC-related pathways, particularly those linked to immune regulation, angiogenesis, and tumor microenvironment remodeling. However, these findings should be interpreted cautiously: HDOCK scores are relative and not equivalent to experimentally measured free energies, and the unusually large negative values reflect the internal scoring function rather than true thermodynamic binding affinities. As such, the docking data are hypothesis-generating and will require confirmation by wet-lab approaches such as RNA pull-down assays, CLIP-seq, or functional knockdown experiments.

## Discussion

Our study provides critical insights into the potential of miR-155 and VEGF as novel biomarkers for CRC, particularly in distinguishing between malignant, benign, and control cases. The demographic analysis revealed that CRC patients had a significantly higher mean age than the benign and control groups. This is consistent with the findings of Akimoto et al. [47], who reported that CRC incidence sharply increases after age 60 due to cumulative genetic mutations and prolonged exposure to carcinogens. However, unlike Cercek et al. [48], who noted an increasing trend in early-onset CRC (patients younger than 50), our study did not observe a similar pattern. This discrepancy may be due to geographical and lifestyle differences, emphasizing the need for regional epidemiological studies to assess CRC risk factors in younger populations [49].

Another important finding was the significant association between smoking and CRC risk. Our data revealed that 64% of CRC patients were smokers, compared to only 37.5% in the benign group and 33% in the control group, with a statistically significant p-value of 0.004. These results align with Hossain et al. [50], who demonstrated that smoking increases CRC risk through the carcinogenic effects of polycyclic aromatic hydrocarbons and nicotine-induced chronic inflammation. Our correlation analysis further supports this association, showing a significant relationship between smoking and both miR-155, suggesting smoking may influence the expression of these biomarkers. The logistic regression analysis identified smoking as an important independent predictor of CRC risk, underscoring its importance as a modifiable risk factor [51].

The significantly higher BMI observed in CRC patients and the higher prevalence of family history of CRC further highlight the multifactorial nature of CRC risk [52]. These findings emphasize integrating modifiable and non-modifiable risk factors into comprehensive CRC risk assessment strategies [53].

One of the key findings of our study was the significantly elevated levels of CA19-9 in CRC patients compared to benign and control groups. This observation agrees with Min et al. [54], who reported that CA19-9 is frequently elevated in CRC but lacks specificity due to its increased levels in pancreatitis and other gastrointestinal disorders. However, our study further underscores the limited diagnostic utility of CA19-9 in early-stage CRC, as its elevation was inconsistent across all cases. This finding contradicts Huang et al. [55], who suggested that CA19-9 has predictive value for metastatic CRC but is less reliable for early-stage detection. These discrepancies highlight the need for a multi-marker approach rather than relying on CA19-9 alone [56].

Similarly, CEA levels did not show a significant difference between CRC, benign, and control groups (p = 0.198), challenging the commonly accepted role of CEA as a standard CRC biomarker. Thomas et al. previously reported that CEA has a sensitivity of 75% in CRC detection, but its performance is significantly reduced in early-stage cases, which aligns with our data [11]. A possible explanation is that CEA is more effective for monitoring disease progression rather than serving as a primary diagnostic tool [57]. Our findings support the growing argument that novel biomarkers are required to improve early CRC detection, particularly in cases where CEA levels remain within the normal range.

Our study identified a significant upregulation of miR-155 in CRC patients (1.8 ± 0.9 fold) compared to the benign (1.3 ± 0.6 fold) and control (0.9 ± 0.5 fold) groups (*p* = 0.003). This finding corroborates the results of Lv et al. [37], who also observed miR-155 overexpression in CRC tissues and serum samples, suggesting its potential role in tumorigenesis. However, unlike prior studies that primarily focused on CRC cases alone [58]. Our study uniquely includes a comparison with benign cases, demonstrating that miR-155 can differentiate between malignant and non-malignant conditions, enhancing its diagnostic specificity.

From a mechanistic perspective, miR-155 regulates CRC progression by targeting PTEN and TGF-*β* signaling pathways, leading to enhanced cell proliferation, invasion, and resistance to apoptosis [36, 59]. Our findings further support this role, as the increased miR-155 levels in CRC patients suggest it contributes to tumor progression by promoting oncogenic pathways [60]. This aligns with Garcia et al. (2021), who reported that miR-155 downregulates tumor suppressors, accelerating CRC growth [61]. Future research should explore whether therapeutic inhibition of miR-155 could serve as a potential intervention for CRC management.

Another novel aspect of our study is the significant elevation of VEGF levels in CRC patients compared to benign and control groups. These results are consistent with Jung et al. [62], who reported that VEGF overexpression is associated with poor CRC prognosis and chemoresistance. However, unlike Lucarini et al. [63], who focused only on late-stage CRC. Our study provides stage-specific quantification, showing that VEGF is already elevated in early CRC, making it a potential candidate for early diagnosis. The substantial difference in VEGF levels between the benign and control groups suggests that VEGF elevation may occur early in the neoplastic process [64].

The underlying mechanism behind VEGF elevation is its role in protecting cancer cells from apoptosis by activating the AKT and NF-κB signaling pathways [65, 66]. By inhibiting apoptotic cascades, VEGF enables CRC cells to survive under stressful conditions, including chemotherapy exposure [67]. Given this mechanism, targeting VEGF with inhibitors could enhance treatment efficacy, making it a diagnostic marker and a potential therapeutic target [68].

A significant strength of our study is the combined analysis of miR-155 and VEGF as a diagnostic panel. ROC analysis revealed that miR-155 alone had an AUC of 0.856, with 84% sensitivity and 86% specificity, which aligns with Zlobec et al. [69]. VEGF demonstrated good discriminatory ability with an AUC of 0.802, sensitivity of 77%, and specificity of 75% [69]. However, when combined, the AUC increased significantly to 0.92 (95% CI: 0.90–0.97), with 91% sensitivity and 92% specificity, surpassing traditional markers like CEA and CA19-9. This improvement highlights the synergistic diagnostic potential of miR-155 and VEGF [70].

Our logistic regression analysis further validates the predictive value of these biomarkers, with miR-155 showing an odds ratio of 2.85 and VEGF an odds ratio of 2.12. The composite score combining these biomarkers exhibited even stronger predictive power, with an odds ratio of 3.25, demonstrating the enhanced value of integrated biomarker approaches. Pearson's correlation matrix revealed important relationships between biomarkers and clinical parameters. miR-155 revealed strong positive correlations with VEGF and the composite score, and moderate correlations with age and smoking. VEGF demonstrated strong correlations with the composite score and smoking and moderate correlations with age and BMI. These interrelationships suggest common biological pathways and risk factors influencing the expression of these biomarkers in CRC [71].

Perhaps most notably, our comprehensive model integrating biomarkers with clinical risk factors (age, smoking, and BMI) achieved exceptional diagnostic performance. The AI-based predictive model enhanced this approach, achieving remarkable performance metrics. These findings demonstrate the potential of machine learning approaches to optimize the integration of diverse parameters for CRC detection, representing a significant advancement over traditional single-marker approaches [72, 73].

The molecular docking analysis revealed strong interactions between MiR-155 and key regulatory proteins, including PTEN, SOCS1, TP53INP1, BCL-6, and IL-13RA1, suggesting a potential role in VEGF signaling and CRC progression [74, 75]. These interactions are particularly relevant to angiogenesis, immune modulation, and tumor microenvironment regulation, all crucial in colorectal cancer detection and therapy [76].

The strong interaction of miR-155 with PTEN (binding energy: − 310.68 kcal/mol) suggests a critical role in modulating the PI3K/AKT pathway, a key signaling cascade that regulates VEGF expression [77]. Soheilifar et al. [70] demonstrated that MiR-155 downregulates PTEN in CRC cells, increasing AKT phosphorylation and VEGF expression and promoting angiogenesis. Our docking results support this by identifying strong hydrogen bonding, ionic, and hydrophobic interactions with key PTEN residues (Lys13, Glu45, Arg72), suggesting a robust inhibitory effect on PTEN function, leading to VEGF upregulation [78].

Similarly, the miR-155_SOCS1 interaction (− 301.94 kcal/mol) underscores the immune-modulatory aspect of this microRNA in CRC. SOCS1 is a suppressor of cytokine signaling that inhibits JAK/STAT activation, a pathway that plays a crucial role in inflammatory cytokine production and VEGF regulation [79, 80]. Adamowicz et al. [81] reported that miR-155 suppresses SOCS1, leading to persistent STAT3 activation, which upregulates VEGF and pro-inflammatory cytokines such as IL-6 and IL-8. Our docking results confirm this interaction through key binding residues (Asp24, Tyr56, and Leu98) with strong stabilizing forces, reinforcing the role of miR-155 in maintaining an inflammatory tumor microenvironment conducive to VEGF-driven neovascularization [82].

The interaction between miR-155 and BCL-6 (− 296.35 kcal/mol) suggests another layer of VEGF regulation via inflammatory pathways. BCL-6 is a transcriptional repressor that regulates NF-κB signaling, a key inflammatory mediator known to enhance VEGF secretion [83]. Zanoaga et al. [84] demonstrated that miR-155 inhibits BCL-6, thereby increasing NF-κB activity and promoting tumor angiogenesis. Our study further supports this hypothesis by revealing strong π-stacking and hydrogen bonding interactions (Arg12, Thr78, and Phe110), reinforcing that MiR-155-driven BCL-6 suppression may enhance CRC's VEGF signaling [85].

The miR-155_IL-13RA1 interaction (− 345.74 kcal/mol) represents another strong regulatory axis in CRC progression. IL-13 signaling has been linked to tumor immune evasion and angiogenesis, as demonstrated by Deng et al. [86], who identified IL-13RA1 as a VEGF modulator in CRC cells. Our docking analysis confirms a robust interaction between miR-155 and IL-13RA1, mediated by Ala22, Gln87, and Asp134, with five hydrogen bonds stabilizing the complex. This suggests that miR-155 may regulate VEGF-mediated tumor progression through IL-13 signaling, providing further evidence of its pro-angiogenic and immune-modulatory effects in CRC [87].

The relatively weaker miR-155_TP53INP1 interaction (− 234.66 kcal/mol) still holds significance in CRC, given TP53INP1's role in apoptosis and cell cycle regulation [88]. While this interaction was moderate compared to other target proteins, our analysis revealed significant binding contributions from Glu31, Ser64, and His101, with four hydrogen bonds stabilizing the complex. TP53INP1 dysregulation has been associated with enhanced tumor survival and resistance to therapy, suggesting that miR-155 suppression of TP53INP1 may provide an additional advantage to VEGF-driven tumorigenesis [89].

Integrating miR-155, VEGF, and immune-regulatory markers (e.g., SOCS1, BCL-6, IL-13RA1) into biomarker-based colorectal cancer detection models could enhance early detection and risk assessment. Given the strong binding affinities observed in our study, targeting MiR-155 or its interactions with PTEN, SOCS1, and BCL-6 may serve as a potential therapeutic intervention for VEGF-driven CRC progression [84, 90]. It should be noted that molecular docking results are based on scoring functions that approximate relative binding propensities rather than absolute biophysical free energies. Therefore, the unusually large negative values reported should be interpreted as comparative rankings among targets, not as true thermodynamic ΔG values. Future wet-lab validation (e.g., RNA pull-down, CLIP-seq) will be necessary to confirm these predicted interactions.

While our study provides strong evidence for miR-155 and VEGF as novel CRC biomarkers, several limitations must be acknowledged. First, we recognize that our study's relatively modest sample size may limit the statistical power and generalizability of the findings. Although the calculated sample size fulfilled the minimum requirement for achieving 80% power at a 95% confidence level, larger multi-center studies are essential to further validate the diagnostic performance of miR-155 and VEGF in diverse populations. Second, while we demonstrated diagnostic potential, future studies should assess whether these markers can predict CRC recurrence and therapy response. Furthermore, although multivariate regression accounted for age and family history, we acknowledge that residual confounding cannot be fully excluded. Future matched case–control studies with larger, multi-center cohorts are needed to confirm the diagnostic accuracy of miR-155 and VEGF under balanced demographic conditions. Also, although the AI model achieved excellent cross-validated performance (AUC 0.96), this finding should be interpreted cautiously given the absence of an external validation cohort. Future work should focus on validating the model in independent, multi-center datasets to assess its generalizability. Finally, a limitation of the docking analysis is that while most protein structures were retrieved from high-resolution PDB crystal structures, some targets (SOCS1 and TP53INP1) lacked experimentally solved structures and were modeled using UniProt/AlphaFold predictions. These predicted models may not fully capture conformational dynamics or ligand-bound states, and therefore, the docking results for these proteins should be interpreted with caution. Another limitation is that our docking simulations treated miR-155 as a rigid structure, which does not fully represent its inherent conformational flexibility. As emphasized by Botti et al. [45], flexible modeling approaches or molecular dynamics simulations are needed to capture biologically relevant RNA dynamics. Accordingly, our docking findings should be interpreted cautiously and considered preliminary until validated by wet-lab experiments.

## Conclusion

In summary, our study provides evidence that miR-155 and VEGF hold promise as synergistic biomarkers for colorectal cancer detection, outperforming conventional markers when combined and further enhanced by integration with clinical risk factors in AI-based models. Importantly, molecular docking results support the biological plausibility of their interaction in CRC progression. However, given the single-center design, modest sample size, and absence of an external validation cohort, these findings should be considered exploratory. Larger, multi-center studies with independent validation must confirm reproducibility, establish clinical utility, and enable translation into precision oncology practice.

## Supplementary Information

Additional file 1

Additional file 2

Additional file 3

Additional file 4

Additional file 5

Additional file 6

## Data Availability

The datasets used and/or analyzed during the current study are available from the corresponding author upon reasonable request.
