# Supplementary material for: Molecular docking insights into miR-155 and VEGF synergy: colorectal cancer detection through AI-enhanced integration of molecular biomarkers and clinical risk assessment
Source: Eur J Med Res. 2025 Dec 4;30:1218. doi: 10.1186/s40001-025-03348-y (PMC12683792; doi:10.1186/s40001-025-03348-y)
Supplement: Supplementary file 6 — Additional file 6 [file 40001_2025_3348_MOESM6_ESM.docx]

**Supplementary Table S1.** Logistic regression model with L2 regularization: partial regression coefficients for predictors of CRC (CRC vs combined benign + control groups)

| Predictor | β (Coefficient) | SE | OR | 95% CI | P-value |
| --- | --- | --- | --- | --- | --- |
| miR-155 | 1.001 | 0.223 | 2.72 | 1.83 – 4.39 | <0.001 |
| VEGF | 0.718 | 0.212 | 2.05 | 1.34 – 3.08 | 0.002 |
| Age (per year) | 0.058 | 0.017 | 1.06 | 1.02 – 1.09 | 0.006 |
| Family history of CRC | 0.936 | 0.421 | 2.55 | 1.08 – 5.63 | 0.031 |

*β = regression coefficient; SE = standard error; OR = odds ratio; CI = confidence interval.*
